# Expanding the Measurement of Suicidal Desire: The Creation and Evaluation of the Suicidal Desire Variability Scale in Three Adult Samples

**DOI:** 10.1007/s10862-026-10286-4

**Published:** 2026-05-01

**Authors:** Raymond P. Tucker, Jessica L. Gerner, Sean M. Mitchell, Nicolas Oakey-Frost, Emma H. Moscardini, Sarah Pardue-Bourgeois, Daniel W. Capron, Ryan M. Hill

**Affiliations:** 1 Department of Psychology, Louisiana State University, 207 Audubon Hall, Baton Rouge, LA 70803, USA; 2 Department of Psychology, The Catholic University of America, Washington, D.C, USA; 3 Department of Psychology, Texas Tech University, Lubbock, TX, USA; 4 University of Texas Health Science Center—San Antonio, San Antonio, TX, USA; 5 McLean Hospital, Belmont, MA, USA; 6 Harvard Medical School, Boston, MA, USA

**Keywords:** Suicide, Ideation, Ecological momentary assessment, Dynamics

## Abstract

This manuscript details a three-study development and preliminary investigation of the Suicidal Desire Variability Scale (SDVS), a self-report retrospective measure of past-week suicidal desire fluctuation. Exploratory factor analyses of the initial 17-item measure performed in 216 (Study 1) undergraduate students experiencing recent suicidal ideation (SI) and confirmatory factor analyses performed in 371 (Study 2) adults with past-week SI demonstrated a two-factor solution of nine original items representing fluctuation in SI (SDVS Dynamic) and minimal change (SDVS Static). Subscales evinced small to moderate correlations with affect and emotional lability as well as no or small relationships with SI severity and its correlates (e.g., wish to live) in Study 2. Study 3 was an ecological momentary assessment investigation of 40 undergraduate students who completed the SDVS following a 10-day ambulatory phase. Retrospective SDVS subscale scores demonstrated moderate correlations with one measure of suicidal desire dynamics assessed across the ambulatory phase.

## Introduction

According to the Centers for Disease Control (CDC), over 13 million adults in the United States (U.S.) reported serious thoughts of suicide in 2022, representing a significant proportion of the population suffering from intense, potentially untreated, psychological distress. Suicidal ideation (SI) is implicated as a causal mechanism in contemporary theories of suicidal behavior, including the Interpersonal Theory of Suicide (Van Orden et al., 2010), the Integrated Motivational Volitional Model (O’Connor & Kirtley, 2018), and the Fluid Vulnerability Theory of suicide (Bryan et al., 2019).

An ever-burgeoning body of basic and applied psychological science has demonstrated (1) the multi-dimensional nature of SI as a construct (e.g., desire for suicide, suicidal intent, etc.; Jobes et al., 2024) and (2) the temporal volatility of these SI dimensions assessed via ambulatory assessment (Coppersmith et al., 2023). Indeed, both passive (i.e., wish to die) and active (e.g., desire to kill oneself) SI appear to be temporally distinct, interrelated, and recursive experiences (Oakey-Frost et al., 2023). Additionally, suicidal *desire* appears to precede suicidal *intent* (i.e., different dimensions of active SI); ratings of suicidal *desire* predict large proportions of change in suicidal *intent* over only a few hours, while the same does not appear to hold true when predicting suicidal desire from suicidal intent (Coppersmith et al., 2023). Kleiman et al. (2017) observed that both suicidal desire and intent vary significantly over time; nearly 30% of ratings in suicidal desire and suicidal intent differ by at least one standard deviation when compared to the previous rating, assessed only hours prior.

Such significant variability in SI-related constructs over the course of hours and minutes is proposed as a significant risk factor for future suicidal behavior both theoretically and empirically (Bryan et al., 2019; Wang et al., 2021). According to the Fluid Vulnerability Theory of suicide, increases in the frequency and magnitude of SI *fluctuations* that significantly diverge from an individual’s “homeostatic equilibrium” may be an important clinical phenotype for future suicidal behavior (Bryan et al., 2019). Empirically, fluctuations in suicidal desire experienced while in psychiatric inpatient care are a better predictor of post-discharge suicidal behavior than average suicidal desire or worst-point suicidal desire during inpatient care (Wang et al., 2021). Indeed, suicidal desire fluctuations seemed particularly important for predicting suicidal behavior compared to fluctuations in suicidal intent or ability to control suicidal impulses (Wang et al., 2021).

These observations suggest that the accurate assessment of variability in suicidal desire, not simply frequency or severity, is important for the assessment of risk for future suicidal behavior (Bryan et al., 2019; Wang et al., 2021). Although the assessment of suicidal desire variability is ideally performed via ambulatory assessment, these procedures may not be feasible in most service delivery systems in which risk information is used to determine outpatient safety and treatment needs (e.g., emergency departments). Furthermore, compliance in the ambulatory assessment of psychological constructs is not guaranteed (Wrzus & Neubauer, 2023). Accordingly, reliable and accurate self-report measurement of variability in suicidal desire may support the assessment of this increasingly important experience in clinical and research efforts. Unfortunately, validated measures of suicide risk assessment instruments exclusively assess frequency and severity of suicidal thoughts and behaviors and not perceived variability/dynamics of these suicide-related experiences (Pollak et al., 2024). Thus, there is a significant need for a retrospective self-report measure of suicidal desire variability that corresponds closely to suicidal desire assessed in real-time and allows for clinical monitoring of the facet of SI in settings in which SI cannot be monitored in real time over the course of a week.

The current series of investigations details the development and initial validation of the Suicidal Desire Variability Scale (SDVS), a self-report measure of past-week suicidal desire variability. The SI facet of suicidal desire, compared to suicidal thinking globally or another facet of SI such as suicidal intent, was chosen given Wang et al.’s (2021) results demonstrating the particularly important contribution of suicidal desire dynamics in prospectively predicting suicide attempts (SAs) in adult psychiatric inpatients. Study 1 detailed the creation of SDVS items and an initial exploratory factor analysis (EFA) in a sample of undergraduate students who had been screened for recent experiences of SI. In Study 2, we conducted a confirmatory factor analysis (CFA) to confirm the factor structure of the SDVS and assess initial convergent and divergent validity of the measure in a sample of U.S. adults who endorsed past-week SI. Finally, Study 3 investigated the test-retest reliability of the SDVS, as well as how well SDVS scores correspond with ambulatory assessment of suicidal desire variability in the week prior to administration to determine the degree of correspondence between past-week retrospective self-report suicide variability and suicidal desire assessed at dozens of time points during the past week.

### Transparency and Openness

The three investigations were not preregistered. Data are not publicly available as informed consent procedures did not indicate that anonymized data would be posted online. However, data are available upon request. Code for statistical analyses for all three investigations can be found here: https://osf.io/2h6qa/?view_only=e0f8629b285c48c9a56c59bd45f437f7. All three investigations were approved by an Institutional Review Board.

## Study 1 Method

### Participants

Participants were 216 undergraduate psychology majors at a large southeastern university who were screened for some level of SI in the prior two weeks as measured by a non-zero score on the first item of the Depressive Symptom Inventory-Suicidality Subscale (DSI-SS; Metalsky & Joiner, 1997), which is further descried in Study 2 below. Participants were 18 to 30 years old (*M* = 19.27, *SD* = 1.36) and identified as primarily women (80.6%), White (48.6%), and heterosexual (56.9%). Most participants endorsed either active or passive SI in the past week (95.3%). See Table 1 for the complete demographics of Study 1 participants.

### Measures

#### Suicidal Desire Variability Scale (SDVS)

The SDVS was created to measure self-reported fluctuations in an individual’s desire to kill themselves over the past week. The set of items tested in Study 1 included 17 items that inquire about the stability or variability in “suicidal desire” or “want” to kill oneself (see Appendix for the full scale). Participants respond to items using a 6-point Likert-type scale ranging from 1 (*strongly disagree*) to 6 (*strongly agree*). The first question in the scale included a response option of “I did not have suicidal thoughts in the past week” to exclude participants without suicidal desire in the last week from the next part of the survey. See the Supplementary Information for a detailed description of the iterative process used to develop SDVS items.

#### Demographics

Participants responded to items that assessed their race, ethnicity, age, sex, gender identity, and sexual orientation.

#### Self-Injurious Thoughts and Behaviors Interview - Revised (SITBI-R)

The SITBI-R (Fox et al., 2020) is a structured clinical interview designed to comprehensively evaluate a wide range of self-injurious thoughts and behaviors. Each section of the SITBI-R begins with screening questions to determine the presence of specific thoughts or behaviors, followed by detailed questions regarding their frequency, context, and characteristics. In the current study, an online self-report form of the SITBI-R was administered. Prior research has demonstrated that the online self-report form of the SITBI-R demonstrates strong psychometric properties (Fox et al., 2020). Two items of the SITBI-R were used to compute frequencies of past-week SI endorsement and SI severity (i.e., passive versus active content) to characterize the sample. History of one or more SAs was assessed using the following single item from the SITBI-R, “Have you ever made an actual attempt to kill yourself in which you had at least some intent to die?” Response options included “yes” and “no.” These data were collected to also help characterize the study sample.

### Procedures

Participants were undergraduate students with SI in the past two weeks, recruited through the SONA system of a large southeastern university. Participants completed a series of questionnaires assessing demographic information as well as mental health- and suicide-related experiences. All participants gave informed consent, received course credit for their participation, and were provided with mental health resources following completion of the study. The appropriate university Institutional Review Board approved all study procedures.

### Analytical Plan

Inattentive or random responding was examined for each participant to determine data quality and inclusion in analyses. We included three attention-check items throughout the survey; participants who missed more than one of these items were excluded from the analyses. For measures that included items requiring reverse coding, we examined response straightlining (i.e., providing the same response on all items for the measure) indicated by *SD* = 0 for their responses on that measure, as well as repeated items (i.e., those who were discrepant in reports of past-week SI); those who straightlined their responses or provided inconsistent responses were removed from analyses. Lastly, we examined the time to complete the survey. Given that participants’ time to complete the survey could be long if they did not close the survey, we examined the lower end of survey completion time. We considered a fast completion time, indicating inattentive responding to be 2 s multiplied by the number of items in the survey.

We used SPSS to examine SDVS item endorsement frequencies and collapsed item endorsement categories if there were response options endorsed with a < 10% frequency to improve EFA model estimation using our specified estimator (DiStefano et al., 2020). Then, we examined inter-item correlations to identify possibly redundant items with higher inter-item correlations (i.e., *r* >.60), as we expected that some items might be highly related due to similar content with minor wording differences between them.

We conducted EFAs using Mplus version 8.11 (Muthén & Muthén, 1998–2024). We modeled the item indicators as categorical, using weighted least squares means and variances (WLSMV) estimation and delta parameterization. We applied an oblique rotation (i.e., Geomin). We tested one and two factor EFAs, evaluating model fit statistics, including chi-square (which can be sensitive to sample size), root mean squared error of approximation (RMSEA), comparative fit index (CFI), Tucker-Lewis Index (TLI), and standardized root mean squared residual (SRMR) relative to recommended cutoff criteria: RMSEA values less than 0.05 indicate excellent fit and 0.05 to 0.08 indicate acceptable fit, CFI values above 0.95, TLI values above 0.95, and SRMR values below 0.08 (Browne & Cudeck, 1993; Hu & Bentler, 1999). Items were also examined to evaluate the interpretability of the factor(s) and item contribution to model fit; items that loaded significantly on more than one factor (i.e., loading > 0.32 and/or *p* <.05) were removed (Osborne & Costello, 2009; Tabachnick & Fidell, 2013). This was done interactively until interpretable and well-fitting models were identified. Following the identification of the final EFA model, McDonald’s ω was calculated.

## Results

As described above, we removed participants with response styles that indicated poor data quality; of the 216 respondents, we retained 195 for analyses. Item response frequencies (see Supplementary Table S1) indicated that item response options of 1 (*strongly disagree*) and 2 (*disagree*), and 5 (*agree*) and 6 (*strongly agree*) should be collapsed given low endorsements at extreme ends of the response scale for nearly all items; notably, items 2 and 17 still had low endorsements even after collapsing those response options and were removed from further consideration. Therefore, for analyses, the response scale ranged from 1 to 4 (recoded as 1 = *Strongly Disagree/Disagree*, 2 = *Somewhat Disagree*, 3 = *Somewhat Agree*, 4 = *Strongly Agree/Agree*). Next, item correlations and descriptive statistics were examined (see Supplementary Table S2). Items 5, 8, 9, and 13 were highly correlated with other items (*r* >.60) and/or had very similar or redundant phrasing with other items that were. Thus, items 2, 5, 8, 9, 13, and 17 were excluded from further analysis.

We conducted EFAs with the remaining 11 SDVS items. Model fit indices and factor loadings are presented in Tables 2 and 3. The model fit for the 11-item one- and two-factor EFAs fit the data poorly. Items 7 and 15 significantly cross-loaded on both factors, so they were removed from the analyses. With the 9 items remaining, EFAs indicated poor model fit for the single factor and adequate model fit for the two correlated factors (*r* = −.61). This 9-item two-factor EFA was interpreted as a *Static* suicidal desire factor (ω = 0.80) and a *Dynamic* suicidal desire factor (ω = 0.80). The 5 items that represent change/variability in suicidal desire (SDVS Dynamic) scored a Coleman–Liau index (a calculator to determine the item readability grade level) of 7.63 and the four items that represent suicidal desire remaining the same or consistent (SDVS Static) scored a 5.99.

### Study 1 Discussion

Results demonstrated adequate initial psychometric properties for a set of two forms of the SDVS distinguished by item direction. Specifically, one 4-item form demonstrated strong reliability with items phrased as suicidal desire remaining static, and another 5-item form demonstrated strong reliability with items phrased as suicidal desire being dynamic. The two forms were highly correlated, bordering on redundancy, highlighting their potential as parallel forms assessing the same construct. Although these results demonstrate promise for the self-report assessment of past-week suicidal desire variability, additional investigation is needed to confirm the factor structure. Additionally, an investigation of the measure’s convergent and divergent validity is important as it is possible the SDVS simply assesses past-week SI intensity, a construct with multiple well-validated measures (Pollak et al., 2024) or other constructs likely closely linked to fluctuations in suicidal desire (e.g., fluctuations in affective states, emotion regulation skills, distress tolerance, etc.).

### Study 2 Method

#### Participants

Participants were 371 U.S adults who endorsed SI in the past week from a single item of the SITBI-R (“When did you most recently have thoughts of killing yourself?” with one response option of, “In the past week”). Participants were 18 to 69 years old (*M* = 34.63, *SD* = 10.49) and identified as primarily women (55%), White (66.8%), and heterosexual (54.2%). Most participants endorsed past-week active SI (74.9%), and a minority endorsed passive SI (21.6%). See Table 1 for the complete demographics of Study 2 participants.

### Materials

#### SDVS

The procedures regarding the SDVS are identical to those described in Study 1. All 17 items from the original scale were administered.

#### Demographics

Identical to Study 1, participants completed measures assessing their age, sex, gender, race, ethnicity, and sexual orientation.

#### SITBI-R

Administration of the SITBI-R was identical to that of Study 1.

#### Past-week SI

Two items were created to assess past-week perceived suicidal desire intensity. As the SITBI-R supports the assessment of the presence of past-week SI and its content (e.g., passive versus active), no ratings of participant perceived intensity of suicidal desire specifically were available from the measure. To determine if participant SDVS scores were correlated with and potentially redundant with measures of past-week suicidal desire severity, items were specifically created to assess these experiences. One item assessed the average suicidal desire intensity over the week (“On average, how intense was your desire to kill yourself over the past week?”), and the other assessed how severe suicidal desire was at its worst point (“At its worst or most intense, how intense was your desire to kill yourself over the past week?”). Both items included a Likert response option from 0 (*not at all intense*) to 4 (*very intense*). Given that these items have not been validated, an existing measure of SI severity was also administered.

#### The Depressive Symptom Inventory-Suicidality Subscale (DSI-SS)

The DSI-SS (Metalsky & Joiner, 1997) is a 4-item self-report assessment of one’s SI severity. Each item is rated on a 4-point scale from 0 (*never*) to 3 (*very often*), capturing the frequency and intensity of suicidal thinking and urges/impulses over the past two weeks. Although most frequently used to rate suicidal thinking over the past two weeks, participants were asked to select answer choices based on their experience in the past week to match the temporal instructions of the SDVS. The DSI-SS has shown excellent psychometric properties, including high internal consistency and good convergent validity (Stanley et al., 2021). The DSI-SS demonstrated adequate internal consistency in the current study (α = 0.75, ω = 0.75).

#### Wish to Live (WTL) and Wish to Die (WTD)

Current WTL and WTD were assessed as convergent validity indicators for the SDVS, given their previously established relationship to SI severity and specifically suicidal desire in ambulatory assessments (Oakey-Frost et al., 2023). The two items administered in the current study mirror those of the Suicide Status Form from the Collaborative Assessment and Management of Suicidality (CAMS; Jobes, 2023). The items read, “Right now I want to live to the following extent” and “Right now I want to die to the following extent,” with response options ranging from 0 (*not at al*l) to 8 (*very much*).

#### Affect Lability Scale-18 (ALS-18)

The ALS-18 (Look et al., 2010) is an 18-item self-report assessment of the extent and nature of an individual’s emotional variability. Participants respond to the items using a Likert-like scale ranging from 0 (*very uncharacteristic of me*) to 3 (*very characteristic of me*). The scale evaluates affective lability across several domains, including anxiety/depression, depression/elation, anger, and anxiety/elation. Higher scores indicate greater emotional instability and fluctuation. The ALS-18 and its subscales have demonstrated high internal consistency, good test-retest reliability, and construct validity (Contardi et al., 2018). In the current study, the ALS-18 was used as a measure of convergent validity, and it was not separated into subscales to reduce the number of analyses. The ALS-18 demonstrated excellent internal consistency (α = 0.92, ω = 0.91).

#### The Difficulties in Emotion Regulation Scale-18 (DERS-18)

The D ERS-18 (Victor & Klonsky, 2016) is an 18-item self-report assessment of individuals’ typical emotion regulation difficulties, which includes 6 factors (i.e., Nonacceptance of Emotional Responses, Difficulties Engaging in Goal-Directed Behavior, Impulse Control Difficulties, Lack of Emotional Awareness, Limited Access to Emotion Regulation Strategies, and Lack of Emotional Clarity). Participants rate each item on a 5-point Likert-like scale ranging from 1 (*almost never*) to 5 (*almost always*). The total score, as well as subscale scores, can be derived to provide a comprehensive assessment of emotion regulation challenges. The DERS-18 has demonstrated strong psychometric properties, including good internal consistency, reliability, and construct validity (Victor & Klonsky, 2016). In the current study, the DERS-18 was used as a measure of convergent validity, and it was not separated into subscales to reduce the number of analyses. The DERS-18 demonstrated adequate internal consistency (α = 0.71, ω = 0.70).

#### The Suicide Cognitions Scale-Revised (SCS-R)

The SCS-R (Bryan et al., 2022) is a 16-item self-assessment of the intensity of suicidal cognitions and beliefs. Items are rated on a 5-point Likert scale ranging from 1 (*strongly disagree*) to 5 (*strongly agree*). The scale evaluates four core dimensions associated with SI, including beliefs about the intolerability of one’s emotional pain, feelings of being unloved or unworthy, perceptions that one’s problems are unsolvable, and feelings of being a burden to others; however, none of the items specifically refer to SI. Only a total score is calculated, with higher scores indicative of more severe suicidal cognitions. The SCS-R has demonstrated excellent psychometric properties, including high internal consistency, good construct validity, and strong predictive validity (Bryan et al., 2020, 2022). The SCS-R was used to test convergent validity in the current study and demonstrated excellent internal consistency (α = 0.94, ω = 0.94).

#### The Distress Tolerance Scale (DTS)

The DTS (Simons & Gaher, 2005) is a 15-item self-report assessment of an individual’s ability to withstand emotional distress. Items are rated on a 5-point Likert-like scale ranging from 1 (*strongly disagree*) to 5 (*strongly agree*). The scale evaluates four dimensions of distress tolerance: Tolerance, Absorption, Appraisal, and Regulation. These dimensions capture an individual’s perceived capacity to endure negative emotional states, their ability to remain focused during distressing experiences, their appraisal of distress as unbearable or unacceptable, and their ability to regulate emotional responses to distress. Higher scores indicate a greater ability to tolerate distress. The DTS has been validated across various populations, demonstrating good internal consistency, and convergent and discriminant validity (Brown et al., 2022; Simons & Gaher, 2005). The DTS was used to assess divergent validity in the current study and was not separated into subscales to reduce the number of analyses. In the current study, the DTS total score demonstrated excellent internal consistency (α = 0.93, ω = 0.93).

#### The Suicide-Related Coping Scale (SRCS)

The SRCS (Stanley et al., 2017) is a 17-item self-report assessment of coping strategies individuals use to manage suicidal thoughts and behaviors and their perceived ability to cope with SI. Items are rated on a 5-point Likert-like scale ranging from 1 (*never*) to 5 (*always*). The scale encompasses various dimensions of coping, such as seeking social support, cognitive reappraisal, problem-solving, and emotion regulation. It has been previously employed to determine if brief interventions for suicide promote coping self-efficacy (Stanley et al., 2017). The SRCS has demonstrated good psychometric properties, including high internal consistency and construct validity (Stanley et al., 2017). Coping self-efficacy was chosen as a potential aspect of divergent validity with SDVS, as variability in suicidal desire should not be redundant with the ability to cope with suicidal desire. Only the 5 items of the SRCS that assess broader beliefs in ability to cope with suicidal thoughts (e.g., “I cannot do anything to control my suicidal thoughts”) were administered in comparison to items specific to coping tools taught in brief interventions (e.g., “I know the nearest hospital or urgent care facility where I can go if I cannot handle my suicidal feelings”) as the scale was administered without explicit provision of or historical experience with brief interventions to increase suicide-related coping self-efficacy. Lower scores on items that assess specific skills taught in interventions (e.g., where local emergency resources are located) could be interpreted as low coping-self efficacy, even when participants scored higher on broader beliefs that they can keep themselves safe from suicide, the construct of interest for divergent validity analyses. The 5 items of the SRCS demonstrated excellent internal validity (α = 0.91, ω = 0.91).

### Procedures

Participants were recruited from the Prolific (www.prolific.com) participant pool. Prolific maintains a database of adults aged 18 years or older who have signed up for the opportunity to participate in research studies via the Prolific website. Studies are made available on the prolific website, and potential participants may decide which studies they wish to participate in. Potential participants were first screened for eligibility, which included being 18 years of age or older, residing in the U.S., and endorsing past-week SI. Participants who were screened as eligible for the study were directed to Qualtrics, an online survey platform, to complete the study.

Participants responded to items that assessed their demographics as well as mental health- and suicide-related experiences. All participants provided informed consent, were compensated by Prolific for completing the study, and received mental health resources upon study completion. All study procedures were approved by the appropriate Institutional Review Board.

### Analytical Plan

First, we examined inattentive or random responding from participants using the same methods described for Study 1. In Study 2, we also collapsed the response options, consistent with Study 1 (recoded as 1 = *Strongly Disagree/Disagree*, 2 = *Somewhat Disagree*, 3 = *Somewhat Agree*, 4 = *Strongly Agree/Agree*). In Study 2, we conducted CFAs using Mplus version 8.11 (Muthén & Muthén, 1998–2024). We modeled the item indicators as categorical, using WLSMV estimation and delta parameterization. We did not correlate error variances; we freely estimated all factor loadings; we constrained the variances of the latent variable(s) to one. We replicated the most interpretable and best-fitting two-factor EFA model using CFA. We evaluated model fit statistics consistent with the Study 1 procedures. Following the final CFA, McDonald’s ω was calculated using SPSS.

To test convergent and divergent validity of the two SDVS factors, bivariate correlations were conducted with measures of affect lability, emotion regulation, and distress tolerance to determine if SDVS scores were related to these constructs as expected. However, if their relationships were extremely strong, this could suggest redundancy and assessment of overlapping constructs (e.g., the SDVS may simply be a measure affect lability, not specific to variability in suicidal desire).

Similarly, bivariate correlations were conducted to determine if SDVS scores relate to past-week SI intensity (DSI-SS total scores, past-week single items of average and worst-point suicidal desire). As with measures of affect lability and distress tolerance, these analyses were conducted to determine if SDVS scores were related to SI frequency and intensity as would be expected, but not so strongly related to suggest redundancy (e.g., the SDVS assesses intensity not variability of SI). Similarly, independent samples *t*-tests were also conducted to determine if SDVS scores differed between the passive and active SI, and between those with and without a history of SA, to test preliminary aspects of clinical utility. Finally, bivariate correlations were conducted to determine if SDVS scores relate to risk factors for STBs (WTL, WTD, suicide cognitions, and suicide coping self-efficacy) to test convergent validity. It would be expected that variability in suicidal desire would be related to constructs previously established to correlate with SI intensity and suicidal desire variability (Moscardini et al., 2023; Oakey-Frost et al., 2023; Stanley et al., 2017).

## Study 2 Results

As described above, we removed participants with response styles that indicated poor data quality. Of the 371 respondents, we retained 361 for analyses. See Supplementary Tables S3 and S4 for the item response frequencies and inter-item correlations for all Study 2 SDVS items.

Using the 9 items from the final Study 1 EFA (5 Dynamic items; 4 Static items), we conducted CFAs, specifying 1- and 2-factor models. As seen in Table 4, these models did not adequately fit the data. This may have been due to the strong latent variable correlation between the two factors (*r* = −.87). Thus, we conducted additional CFAs with the 5 Dynamic items specified as 1 factor and the 4 Static items specified as 1 factor, in separate models. As shown in Table 4, these 1-factor models fit the data very well and demonstrate strong internal consistency (ω = 0.88 for both factors). See Supplementary Table S5 for item factor loadings. These models provided potential support for separate parallel assessment forms, one to assess Static suicidal desire and another to assess Dynamic suicidal desire.

As an additional test of the potential parallel nature of the two forms, a paired samples *t*-test of item averages on each form was conducted. Participants, on average, scored higher on the reverse-coded items of the Static form (*M* = 2.69, *SD* = 1.00) compared to the Dynamic form (*M* = 2.40, *SD* = 0.94; *t*(360) = −7.95, *p* <.001, *d* = 0.70).

### Convergent/Divergent Relationships with Affect and Emotion Instability

As seen in Table 5, SDVS Dynamic scores were positively correlated with affect lability (moderate effect size; Cohen, 1988) and emotion dysregulation (small effect size) and negatively correlated with the ability to tolerate distress (small effect size). SDVS Static scores were negatively correlated with affect lability, with a small effect size, and not significantly related to emotion regulation and distress tolerance.

### Convergent/Divergent Relationships with STBs

As seen in Table 5, SDVS Dynamic was positively related to past-week worst point suicidal desire with a small effect size, but it was not significantly related to SI intensity total score and past-week average suicidal desire. SDVS Static was positively related to SI intensity and past-week average suicidal desire with small effect sizes, but was not significantly related to past-week worst point suicidal desire.

Both SDVS Static and Dynamic scores were not significantly different between those who endorsed past-week passive (*M* = 9.30, *SD* = 3.72 and *M* = 11.87, *SD* = 12.10) versus active SI (*M* = 9.18, *SD* = 4.03 and *M* = 12.10, *SD* = 4.70; *t*(346) = 0.20, *p* =.843, *d* = 0.03 and *t*(346) = − 0.39, *p* =.70, *d* = −0.02, respectively). SDVS Static scores were elevated in those with (*M* = 10.04, *SD* = 3.98) versus without (*M* = 8.78, *SD* = 3.94) a SA history (*t*(259.05) = −2.84, *p* =.005, *d* = 0.32). SDVS Dynamic scores were not significantly different in those with (*M* = 11.65, *SD* = 4.64) versus without (*M* = 12.16, *SD* = 4.73) a SA history (*t*(359) = 0.99, *p* =.324, *d* = −0.10).

### Convergent/Divergent Relationships with STB Risk Factors

As seen in Table 5, both SDVS Dynamic and Static scores were related to WTD and WTL ratings, with SDVS Static scores demonstrating moderate effect sizes and SDVS Dynamic demonstrating small effect sizes. SDVS Dynamic demonstrated non-significant relationships with suicide cognitions and suicide-related coping self-efficacy, whereas SDVS Static demonstrated small relationships with these constructs.

## Study 2 Discussion

The CFA results demonstrate modest support for the factor structure and strong support for the internal consistency of two forms of the SDVS separated by item wording (i.e., Static vs. Dynamic). The parallel or alternate form hypothesis of the SDVS items posed after the EFAs in Study 1 was not supported in Study 2, as the forms demonstrated significantly different mean item scores despite a large correlation between the subscales. A commonly held criterion for parallel forms, having relatively equal means (Gulliksen, 1950), was clearly not met in Study 2. This result suggests that the forms, despite their development to assess the same construct, may be assessing slightly discrepant constructs. Convergent and divergent validity analyses also supported this interpretation. Although these analyses demonstrated that neither form appears to redundantly capture similar information (e.g., SI severity or frequency), discrepant patterns of convergent validity were demonstrated. Namely, SDVS Static demonstrated a stronger pattern of expected convergent validity indicators (e.g., relationships with past week SI and STB risk factors) compared to SDVS Dynamic. However, SDVS Dynamic was more uniformly related to constructs related to affect and emotional lability compared to SDVS Static. These results could be interpreted as SDVS forms not simply assessing affect lability or SI frequency and intensity, but that SDVS Static items may assess suicide-related thinking variability more closely compared to SDVS Dynamic scores, given their more consistent relationship with SI severity. This interpretation is certainly preliminary, and results warrant replication, particularly with objective measurement of past-week suicidal desire variability using ambulatory assessment of moment-to-moment SI.

## Study 3 Methods

### Participants

A total of 43 undergraduate students completed baseline assessments. Of these participants, *n* = 2 did not complete any ambulatory assessments, and an additional *n* = 1 individual did not return for T2 assessment; thus, their data were not analyzed. Table 1 provides a detailed description of the analyzed sample of 40 undergraduate students ages 18–22 years (*M* = 19.58, *SD* = 1.39). Participants identified as predominantly women (80%), White (45%) or Black (25%), and heterosexual (57.5%) or bisexual (27.5%).

### Materials

#### SDVS

The administration of the SDVS was identical to that of Studies 1 and 2.

#### Demographics

Identical to Studies 1 and 2, participants responded to items that assessed their age, sex, gender, race, ethnicity, and sexual orientation.

#### Ambulatory Items

During the ambulatory phase of this study, participants responded to two items presented with a visual analogue slider, representing suicidal desire: “right now, I want to kill myself” and “right now, I have an intense desire to kill myself.” Participants chose their value up to two decimal places (e.g., 0.23 or 0.72), using a sliding scale with anchors of 0 (*Not at All*) to 1 (*Extremely*). These items were adapted from past ambulatory studies assessing state SI (e.g., Kleiman et al., 2017; Hallensleben et al., 2019) and selected to represent two items that assess the same construct of suicidal desire, albeit with slightly discrepant wording. Research highlights the importance of how subtle language variations in items assessing similar SI-related constructs can influence participant responses (Ammerman et al., 2021). To examine the relationship between the SDVS and the ambulatory assessment of suicidal desire variability over the past week, the authors utilized multiple items. This approach aimed to assess whether relationships were consistent across differently phrased items measuring the same construct of suicidal desire.

### Procedures

The appropriate Institutional Review Board approved all study procedures. Undergraduate students at a large southeastern university were recruited through the university’s SONA system to participate. Those who reported SI within the past two weeks (i.e., a non-zero response on the first item of the DSI-SS) received an email invitation to participate in the study. Students interested in participating followed a link in the invitation email to the SONA signup page, where they scheduled a meeting with a graduate student research assistant.

Participants met with a graduate research assistant via video conference (i.e., Zoom), where they provided informed consent and received information regarding the study procedures. During the first session, participants completed demographic measures and the SDVS (T1) through Qualtrics. Copies of the informed consent and mental health resources were emailed to each participant after the baseline session. A suicide safety plan (Stanley et al., 2018) was also completed at the end of the baseline session to help increase participant safety during the ambulatory period and determine the immediate need for services.

### Ecological Momentary Assessment (EMA) Procedure

Following the completion of baseline survey measures (e.g., demographics), participants downloaded the PIEL EMA application (Jessup et al., 2012) to their smartphones. They were instructed on how to use the application through an example survey. For ten days, participants responded to brief surveys five times daily via PIEL. These signal-contingent, pseudorandom surveys were deployed within three-hour time blocks (e.g., 9 am to 12 pm) between 9 am and 11:59 pm, covering questions related to SI and its risk and protective factors (only items that represent suicidal desire were analyzed in the current investigation). Reminder emails were sent immediately after the initial meeting and every other day throughout the study period. Participants could respond to these emails if they encountered technical issues.

### Follow-Up Procedure

After the ten-day ambulatory assessment period, participants had a brief follow-up meeting with a research assistant over Zoom to transfer their data, complete the SDVS for a second time (T2), and arrange for compensation. Due to scheduling variability, the SDVS was not always collected immediately following the assessment period.

### Analytical Plan

To determine the test-retest reliability of the SDVS, bivariate correlations were conducted between the two forms of the measure completed at T1 and T2. To provide an estimate of average variability in the two suicidal desire ambulatory items over the 10-day ambulatory phase, the root mean square of successive differences (RMSSD) for each item was calculated. Higher RMSSD values indicate greater variability from one assessment to the next. Additionally, intra-class correlations (ICCs) were calculated, with values ranging from 0 to 1; values closer to 1 indicate that a greater proportion of the total variance is attributable to between-person differences (1 – ICC reflects the proportion attributable to within-person variability). The percent of assessments in which scores on ambulatory items changed by 1 *SD* or greater was also computed to represent the intensity of suicidal desire variability when the assessment of the construct changed between consecutive assessments. Bivariate correlations were conducted between RMSSD values, percent of responses in suicidal desire ambulatory items that changed by 1 *SD* or greater, and SDVS Dynamic and Static forms completed at T2 to determine if retrospectively self-reported suicidal desire variability correlated with suicidal desire variability during the ambulatory period.

## Study 3 Results

Participants completed an average of 76.2% of surveys (*M* = 38.1; *SD* = 10.7; Observed Range = 10–50 out of 50 possible) over the 10-day ambulatory assessment period. The final number of surveys was *k* = 1564. The number of surveys completed was not significantly related to SDVS subscale scores at baseline or follow-up (*p*s > 0.26). On average, there was a 3.58-day lag (*SD* = 3.28) between the last ambulatory assessment and the completion of the SDVS at T2.

For the *desire* for suicide variable, the average response was 0.15 (*SD* = 0.21, Skew = 1.51, Kurtosis = 1.55, Range = 0–1). For the *want* for suicide variable, the average response was 0.17 (*SD* = 0.22, Skew = 1.42, Kurtosis = 1.22, Range = 0–1). Regarding variability statistics of the EMA items, the average RMSSD was 0.11 for desire for suicide (Range = 0.004 – 0.32), and 0.13 for want for suicide (Range = 0.01 – 0.34). The ICC value for desire for suicide was 0.65 (CI = 0.55 – 0.76) and for want for suicide was 0.62 (CI = 0.52 – 0.73). The average of participant percentages of ratings that changed by at least 1 *SD* from one timepoint to the next was 25.20% for desire and 27.12% for want.

As demonstrated in Table 6, T1 and T2 SDVS Static were correlated with a large effect size, as were T1 and T2 SDVS Dynamic. Cross-form relationships between the subscales (e.g., T1 SDVS Dynamic and T2 SDVS Static) ranged from *r* = −.47 – − 0.56.

Both T2 SDVS Dynamic and Static were correlated with both suicidal desire ambulatory items, with moderate effect sizes. Neither SDVS form was significantly correlated with the percent of consecutive periods in which ambulatory items changed by 1 *SD* or more.

### Post-Hoc Analyses

To support the potential clinical utility of the SDVS forms and their ability to accurately represent past-week suicidal desire variability, the correlations between each of the SDVS items and the RMSSD values and percent of responses in suicidal desire ambulatory items that changed by 1 *SD* or greater were explored. These analyses could support initial considerations of adding one or more items to clinical risk assessment and epidemiologic investigations of SI, particularly when assessment time is limited.

As seen in Supplementary Table 6, no SDVS items across the two forms were related to the percentage of suicidal desire change of 1 *SD* or more, and five SDVS items were not significantly related to the RMSSD scores. One item (“How much I wanted to kill myself went up and down a lot”) was positively and moderately correlated with the “right now, I want to kill myself” item, but not significantly related to the “right now, I have an intense desire to kill myself” item. Three SDVS items showed consistent, moderate to strong relationships with both ambulatory items. The item “How much I wanted to kill myself did not change very often” was negatively related to RMSSD values of both ambulatory items, while “I frequently switched from wanting to kill myself to not wanting to kill myself” and “Many times, I quickly went from not wanting to kill myself to really wanting to kill myself” were positively associated with both ambulatory items RMSSD values.

## Study 3 Discussion

Consistent with the study’s objectives, the SDVS demonstrated moderate correlations with ambulatory assessment-derived measures of suicidal desire variability, suggesting that retrospective self-reports may be able to capture meaningful aspects of SI dynamics when ambulatory assessment is not feasible. Further, post-hoc analyses demonstrated that three items of the SDVS evinced the most robust association with suicidal desire dynamics to be further explored in subsequent research. It should be noted, however, that relationships between retrospective self-report SDVS scores and RMSSD values from the ambulatory assessment period were not strong in effect size, suggesting that ambulatory assessment of suicidal desire dynamics remains a more accurate strategy for assessing changes in SI over time and underscores research demonstrating that retrospective self-reports do not fully capture in-the-moment experiences of suicidal thinking (Gratch et al., 2021; Shin, Gratch & Cha, 2025).

## General Discussion

The current set of investigations sought to develop and test a self-report measure of past-week suicidal desire variability. Research indicates that variability of suicidal desire, not frequency of experience or severity, may be the strongest prospective predictor of SA in adult psychiatric inpatients; thus, the assessment of variability in this SI content is clinically important (Wang et al., 2021). A self-report measure that demonstrates reasonable accuracy in assessing recent variability in suicidal desire is poised to support research efforts to better understand who experiences highly variable suicidal desire and why, which is both critical for continued theory development about who dies by suicide and when. Similarly, the creation of an accurate self-report measure may aid in clinical risk assessment as validated self-report measures and semi-structured risk assessment tools assess the frequency and severity of multiple contents of SI (desire for suicide, suicidal intent, etc.) but do not currently assess for variability in these thoughts (Pollak et al., 2024).

EFAs completed in college students and CFAs completed in adults recruited online—the majority of whom endorse some SI in the past week—indicated that the original 17 items developed are best distilled down to two related constructs: one form containing four items phrased as suicidal desire remaining static throughout the past week, and another five-item form with items regarding the experience of dynamic suicidal desire experienced in the past week. Both forms demonstrated similarly strong internal consistency in both samples.

Despite both forms being from the same initial item pool developed to assess the same construct, their parallel nature is unlikely, meaning they should not be used as separate, interchangeable measures. In Study 2, SDVS Static and Dynamic were highly correlated (*r* = −.71; latent variable correlation of − 0.87), but their average item means differed significantly. There are no formal criteria for when forms are adequately parallel; however, parallel forms should return relatively equal item averages when assessed in the same set of participants.

The notion that the two SDVS forms are parallel, and thus equally assess the same construct, was further refuted in convergent and divergent validity analyses. In Study 2, SDVS Dynamic scores were correlated with all constructs related to affect instability, emotion regulation concerns, and distress intolerance, but were not significantly related to almost all aspects of past-week SI. Conversely, items that represented consistent suicidal desire over the past week (SDVS Static) were correlated to all but one aspect of past-week SI and were related to affect instability but not emotion regulation concerns or distress tolerance. The validity indicators of the forms are promising, as neither form demonstrates redundant correlations with scales whose item structure was reviewed during initial SDVS item development (ALS) or with measures of SI frequency and severity experienced within the same timeframe (DSI-SS). However, the discrepant convergent validity correlations suggest that the forms may not be assessing the same construct.

These elements of convergent and divergent validity are important for evaluating the psychometric properties of the SDVS forms. However, their relationship to real-time assessments of suicidal desire variability may provide the strongest evidence of the measures’ validity and clinical utility. Despite previous results that demonstrate that adults tend to overestimate their prospective likelihood of experiencing suicidal desire (Coppersmith et al., 2024) and retrospective assessment of SI severity may not perfectly align with ambulatory assessment (Gratch et al., 2021; Shin, Gratch & Cha, 2025), it appears that college students with SI can reasonably report how variable their suicidal desire was in the past week. Additionally, scores on three specific items of the SDVS were particularly consistent with suicidal desire variability assessed in real time (“*How much I wanted to kill myself did not change very often*” *I frequently switched from wanting to kill myself to not wanting to kill myself*” and “*Many times*, *I quickly went from not wanting to kill myself to really wanting to kill myself*”). These three items evidenced moderate to strong correlations with two different ambulatory assessment items of suicidal desire. It should be noted that no SDVS items correlated with the percent of instances in which ambulatory assessed suicidal desire changed by 1 *SD* or more. This could mean that SDVS items assess variability in the past week but *not* how intense shifts in variability are.

Interpretations of the moderate accuracy of self-reported suicidal desire are made very tentatively, as Study 3 correlations between suicidal desire variability derived from ambulatory and self-report assessment were conducted in a small sample (*n* = 41). Additionally, correlations were moderate in size, certainly not representing a high degree of accuracy. Similarly, average RMSSD found in Study 3 were visibly lower than what has been found in adult inpatient samples and in those recently treated for suicidal thoughts and behaviors (Kleiman et al., 2017; Wang et al., 2021). It is possible that the ability to accurately report variability in past-week suicidal desire may be moderated by variability itself, in which people who experience higher variability in their suicidal desire demonstrate less accurate retrospection of this variability than those whose desire remained relatively consistent. Finally, another possibility is that the ambulatory assessment period may have enhanced participants’ ability to reflect on their variability in suicidal desire, which in turn could have impacted SDVS scores. Alternatively, the creation of the suicide safety plan at the end of baseline data collection could have helped participants understand their triggers for suicide (step 1 in suicide safety planning) and thus better monitor these triggers and subsequent suicidal thoughts during the ema phase. These possibilities cannot be refuted with data from the current series of investigations, and future research should consider psychological and methodological factors that influence the relationship between SDVS scores and suicidal desire dynamics assessed during ambulatory assessment in future research.

### Limitations

The results of this study should be considered in light of its strengths and limitations. Participants in Study 1 were recruited based on reporting SI in the two weeks before the start of the semester, yet they may have participated later in the semester. As a result, a small percentage did not report past-week SI during the study. In Study 3, there was an average of 3.58 days between the last ambulatory assessment of suicidal desire and the completion of T2 SDVS measurement. Regarding the SDVS measure itself, the SDVS does not differentiate between different suicidal cognitions, such as suicidal intent versus suicidal desire. Recent findings indicate that suicidal desire and suicidal intent vary on different timescales (Coppersmith et al., 2023). As such, future research may need to examine whether SDVS results differ when participants respond regarding different types of SI. Finally, the creation of the SDVS was predicated on the particularly important relationship between suicidal desire dynamics and future suicidal behavior (Wang et al., 2021), but the current study did not explicitly test the prospective predictive validity SDVS scores. Given the initial support for the reliability and validity of the SDVS, studies that investigate the measure’s clinical utility are a logical next step.

### Constraints on Generalizability

Regarding study samples, each of the studies included a strong representation of sexual minority (i.e., non-heterosexual) and racial and ethnic minority populations, a clear strength compared to average minority representation in contemporary psychological research (Wilson, 2024). Even so, two of the studies consisted of predominantly cisgender women (Studies 1 and 3), and one study drew upon a predominantly White sample (Study 2), potentially limiting generalizability. Additionally, Studies 1 and 3 drew on college student samples, which may also limit generalizability, as college students are not fully representative of the overall population. Additionally, study samples were identified based on the presence of recent SI, which may limit the inclusion of those with intermittent SI. Thus, individuals who experience infrequent, but potentially intense SI, may be underrepresented in the sample. Future research should consider evaluating suicide variability over longer durations to better reflect individuals who experience SI infrequently. Finally, the grade-estimated reading level of the SDVS subscales, ranging from 5.99 (SDVS Static) to 7.63 (SDVS Dynamic), may limit generalizability to those with lower reading abilities. Estimates for the average reading level of adults in the U.S. range from 6th to 8th grade (National Center for Education Statistics, 2024; Rothwell, 2020), indicating that a significant number of U.S. adults may struggle to comprehend items in both subscales. Future research should consider and attempt to empirically study this concern.

## Conclusions

The current studies developed and tested a self-report measure of past-week suicidal desire variability. Given research that indicates the variability of suicidal desire, not frequency of experience or severity, may be the strongest prospective predictor of SA in adult psychiatric inpatients (Wang et al., 2021), a self-report measure of variability in suicidal desire can support efforts to better understand this phenomenon. The current studies provide preliminary support for a measure of variability in suicidal desire evaluated across three samples of adults with recent SI. Results should be interpreted with caution due to the use of non-clinical samples, inconsistent model fit seen between Studies 1 and 2, and the need to collapse item response categories in EFA analyses in Study 1. Thus, at this time, the SDVS should not be used in clinical settings; however, preliminary evidence of the scale’s reliability and validity provides a clear rationale for future study. To continue to best understand the factor structure of the SDVS in subsequent research, it is recommended that future research include all 17 of the initial items using the original 6-point Likert scale to further confirm (or refute) the modest support for the two-factor structure seen in this series of investigations.

## Supplementary Material

supplemental material

**Supplementary Information** The online version contains supplementary material available at https://doi.org/10.1007/s10862-026-10286-4.

## Figures and Tables

**Table 1:** Demographic Characteristics of Participants from Studies 1, 2, and 3

	Study 1 (*N* = 216)		Study 2 (*N* = 371)		Study 3 (*N* = 40)	
	*n*	%	*n*	%	*n*	%
Sex						
Male	42	19.4	166	44.7	5	12.5
Female	174	80.6	204	55	35	87.5
Gender						
Man	41	19	157	42.3	4	10
Woman	165	76.4	180	48.3	32	80
Transgender	3	1.4	5	1.3	1	2.5
Non-binary	7	3.2	27	7.3	3	7.5
Not listed	NA	NA	2	0.5	0	0
Sexual Orientation						
Straight	123	56.9	201	54.2	23	57.5
Gay	6	2.8	7	1.9	0	0
Lesbian	8	3.7	14	3.8	1	2.5
Bisexual	53	24.5	109	29.4	11	27.5
Not sure/Not listed	26	12	39	10.5	5	12.5
Race/ethnicity						
White	105	48.6	248	66.8	18	45
African American/Black	51	23.6	39	10.5	10	25
Hispanic/Latino	14	6.5	25	6.7	4	10
Asian/Asian American	27	12.5	35	9.4	3	7.5
American Indian or Alaskan Native	0	0	3	0.8	0	0
Biracial	18	8.3	19	5.1	4	10
Not listed	1	0.5	2	0.5	1	2.5
Suicidal Ideation						
None in past week	10	4.6	13	3.5	NA	NA
Past week passive suicidal ideation	91	42.1	80	21.6	NA	NA
Past week active suicidal ideation	115	53.2	278	74.9	NA	NA
Suicide Attempts						
No	142	65.7	240	64.7	NA	NA
Yes	74	34.3	131	35.3	NA	NA

**Table 2:** Study 1 SDVS Exploratory Factor Analyses Model Fit Indices

Model	Number of Free Parameters	Model Fit Statistics				
		X^2^, df, *p*	RMSEA, (90% CI), *p*	CFI	TLI	SRMR
11 SDVS items, 1 factor	11	208.94, 44, < 0.001	0.14, (0.12, 0.15), < 0.001	0.90	0.88	0.11
11 SDVS items, 2 factors	21	99.91, 34, < 0.001	0.10, (0.08, 0.12) < 0.001	0.96	0.94	0.06
9 SDVS items, 1 factor	9	164.23, 27, < 0.001	0.16, (0.14, 0.19), < 0.001	0.88	0.84	0.13
9 SDVS items, 2 factors	17	46.89, 19, < 0.001	0.09, (0.06, 0.12), 0.028	0.98	0.96	0.06

*SDVS* = Suicidal Desire Variability Scale, *RMSEA* = root mean squared error of approximation, *CFI* = comparative fit index, *TLI* = Tucker-Lewis Index, *SRMR* = standardized root mean squared residual

**Table 3:** Study 1 SDVS Exploratory Factor Analyses Item Loadings

SDVS Item	11-item Models			9-item Models		
	One Factor	Two Factors		One Factor	Two Factors	
		1	2		1	2
1	0.40*	0.15	0.58*	0.41*	0.14	0.58*
3	−0.63*	0.51*	−0.20	−0.65*	0.52*	−0.21*
4	−0.69*	0.83*	0.08	−0.67*	0.82*	0.08
6	−0.83*	0.90*	−0.00	−0.82*	0.90*	−0.00
7	0.82*	−0.45*	0.47*	----	----	----
10	0.68*	−0.06	0.68*	0.64*	−0.04	0.66*
11	0.78*	−0.10	0.75*	0.77*	−0.10	0.75*
12	−0.71*	0.72*	−0.07	−0.73*	0.73*	−0.09
14	0.76*	−0.00	0.83*	0.75*	−0.01	0.82*
15	−0.60*	0.34*	−0.33*	----	----	----
16	0.56*	0.25*	0.83*	0.59*	0.22*	0.84*

*SDVS* = Suicidal Desire Variability Scale, 11-item two-factor model factor correlation *r* = −.62, *p* < .05; 9-item two-factor model factor correlation *r* = −.61, *p* < .05; 9-item Omega Internal Consistency = .82, Dynamic Items Omega Internal Consistency = .78, Static Omega Internal Consistency = .80.

* *p* < .05

**Table 4:** Study 2 SDVS Confirmatory Factor Analyses Model Fit Indices

Model	Number of Free Parameters	Model Fit Statistics				
		X^2^, df, *p*	RMSEA, (90% CI), *p*	CFI	TLI	SRMR
9 SDVS items, 1 factor	36	417.65, 27, < 0.001	0.20, (0.18, 0.22), < 0.001	0.95	0.93	0.07
9 SDVS items, 2 factors	37	320.85, 26, < 0.001	0.18, (0.16, 0.20), < 0.001	0.96	0.95	0.05
5 SDVS items, 1 Factor Dynamic Model	20	13.31, 5, 0.021	0.07, (0.02, 0.11), 0.210	> 0.99	> 0.99	0.01
4 SDVS items, 1 Factor Static Model	16	15.48, 2, < 0.001	0.14, (0.08, 0.20), 0.009	> 0.99	0.99	0.01

*SDVS* = Suicidal Desire Variability Scale, *RMSEA* = root mean squared error of approximation, *CFI* = comparative fit index, *TLI* = Tucker-Lewis Index, *SRMR* = standardized root mean squared residual

**Table 5:** Study 2 Convergent and Divergent Correlations between the SDVS, Affect and Emotional Lability, Distress Intolerance, and Suicide Risk Factors

Variable	1	2	3	4	5	6	7	8	9	10	11	12
1. SDVS Static	---											
2. SDVS Dynamic	−0.74***	---										
3. Suicide Cognitions	0.22***	−0.04	---									
4. Suicide Ideation	0.18***	−0.07	0.52***	---								
5. Suicide-Related Coping Self-Efficacy	−0.20***	0.04	−65***	−0.46***	---							
6. Past Week Average Suicidal Desire	0.13*	0.05	0.55***	0.65***	−0.44***	---						
7. Past Week Worst Point Suicidal Desire	0.01	0.14*	0.52***	0.63***	−0.37***	0.76***	---					
8. Wish to Live	0.35***	−0.15**	0.67***	0.57***	−0.51***	0.56***	0.47***	---				
9. Wish to Die	−0.33***	0.22***	−0.62***	−0.48***	0.48***	−0.39***	−0.32***	−0.71***	---			
10. Affect Lability	−0.20***	0.40***	0.18***	0.09	−0.13*	0.24***	0.30***	0.00	0.08	---		
11. Emotion Dysregulation	0.04	0.13***	0.45***	0.27***	−0.39***	0.27***	0.27***	−0.28***	0.29***	0.48***	---	
12. Distress Intolerance	0.01	−0.15**	−0.45***	−0.31***	0.41***	−0.40***	−0.38***	0.22***	−0.30***	−0.49***	−0.68***	---
*M*	9.23	11.98	30.05	4.91	12.56	1.55	2.10	3.50	4.23	23.93	49.63	10.00
*SD*	3.99	4.70	15.96	1.97	5.13	1.00	1.13	2.20	2.02	11.28	12.55	3.37
*Skewness*	0.23	0.10	0.16	0.57	−0.52	0.54	−0.11	0.13	−0.01	−0.11	0.02	0.26
*Kurtosis*	−1.19	−1.20	−0.63	1.27	−0.55	−0.03	−0.69	−0.90	−0.71	−0.60	−0.51	−0.64

* *p* < .05,

** *p* < .01,

*** *p* < .001.

SDVS = Suicidal Desire Variability Scale

**Table 6:** Study 3 Correlations between T1 and T2 SDVS and Measures of Suicidal Desire Variability During EMA Assessment Period

Variable	1	2	3	4	5	6	7	8
1. T1 SDVS Static	----							
2. T1 SDVS Dynamic	−0.51***	----						
3. T2 SDVS Static	0.71***	−0.47***	----					
4. T2 SDVS Dynamic	−0.55***	0.73***	−0.71***	----				
5. Desire RMSSD	−0.10	0.17	−0.31*	0.37*	----			
6. Want for Suicide RMSSD	−0.29	0.26	−0.35*	0.41**	0.78***	----		
7. 1 *SD* Change in Desire	0.02	−0.08	−0.03	−0.15	0.11	0.10	----	
8. 1 *SD* Change in Want	−0.03	0.12	−0.10	0.09	0.29	0.11	0.44**	----
*M*	9.41	9.74	9.93	10.63	0.12	0.13	25.21	26.97
*SD*	4.48	4.96	4.81	5.30	0.08	0.09	13.09	12.54
*Skewness*	0.37	0.75	0.05	0.41	0.60	0.72	0.40	−0.23
*Kurtosis*	−1.32	−0.69	−1.65	−1.42	−0.47	−0.05	−0.18	−0.90

* *p* < .05,

** *p* < .01,

*** *p* < .001.

T1 = Timepoint 1, T2 = Timepoint 2, *SDVS* = Suicidal Desire Variability Scale, *RMSSD* = root mean square of successive differences

## Public Significance Statement:

Adults with past-week suicidal thinking can, with a moderate degree of accuracy, report the extent to which their suicidal thoughts were dynamic or static over the past week. This measure shows promise for integration into clinical and research initiatives to assess the important construct of suicidal desire dynamics when multiple assessments during the week are not feasible.

## Appendix

### Suicidal Desire Variability Scale

For some people, thinking about killing themselves come and go. On the other hand, some people’s thoughts of suicide do not change much (they always want to kill themselves, never want to kill themselves, or somewhere in-between but it does not change over time).

Below you will see sentences and pictures that describe how some people experience thoughts of suicide and how they change from moment to moment over time. Please read each item carefully and rate the extent to which you agree with it using the 1–6 scale provided.

Please rate the extent to which you agree with the following items.

1 = Strongly Disagree, 2 = Disagree, 3 = Somewhat Disagree, 4 = Somewhat Agree, 5 = Agree, and 6 = Strongly Agree.

**** The first question in the scale included a response option of, “I did not have suicidal thoughts in the past week” in order to skip participants without suicidal desire in the last week to the next part of the survey******.

For the following questions, please focus on any thoughts of suicide you have had *in the past week*.

1. How much I wanted to kill myself went up and down a lot^D^.
2. My thoughts of suicide came and went.
3. How much I wanted to kill myself did not change very often^s^.
4. How much I wanted to kill myself stayed about same from one hour to the next^s^.
5. From one hour to the next, my thoughts of suicide changed.
6. From one hour to the next, my thoughts of suicide did not change^s^.
7. From one hour to the next, how much I wanted to kill myself changed a lot.
8. From one hour to the next, my desire to kill myself stayed about the same.
9. From one hour to the next, the extent to which I wanted to kill myself stayed about the same.
10. How much I wanted to kill myself changed multiple times from one hour to the next^D^.
11. My desire to kill myself was mostly unpredictable because it changed so quickly^D^.
12. My desire to kill myself was fairly predictable because it did not change much^s^.
13. One minute I really wanted to kill myself and the next minute I didn’t.
14. I frequently switched from wanting to kill myself to not wanting to kill myself^D^.
15. I rarely shifted back and forth from wanting to kill myself to not wanting to.
16. Many times, I quickly went from not wanting to kill myself to really wanting to kill myself^s^.
17. There were times when I really wanted to kill myself then hours later I didn’t.

#### Scoring Guide.

^D^Items retained on the form regarding dynamics or variability in suicidal desire, ^s^Items retained on the form regarding suicidal desire remaining static or consistent, grey items are those that were excluded from the factor analysis models. Items within each subscale are summed to create the SDVS Static and SDVS Dynamic subscales. In initial analyses, the response scale was recalculated due to insufficient endorsement and thus ranged from 1 to 4 (recoded as 1 = Strongly Disagree/Disagree, 2 = Somewhat Disagree, 3 = Somewhat Agree, 4 = Strongly Agree/Agree). Given these initial validation results, it is recommended that researchers administer all 17 items, as the two-subscale model identified was inconsistent across studies 1 and 2; thus, the scale’s factor structure may differ in subsequent administrations. As Items 3, 14, and 16 demonstrated the strongest relationships with suicidal desire assessed in real-time, it is recommended that at least these three items be considered in subsequent research studies.
