# Supplementary material for: Expanding the Measurement of Suicidal Desire: The Creation and Evaluation of the Suicidal Desire Variability Scale in Three Adult Samples
Source: J Psychopathol Behav Assess. Author manuscript; Available in PMC 2026 May 22. (PMC13186210; doi:10.1007/s10862-026-10286-4)
Supplement: supplemental material [file NIHMS2174657-supplement-supplemental_material.docx]

**Expanding the measurement of suicidal desire: The creation and evaluation of the Suicidal Desire Variability Scale in three adult samples**

**Detailed Information Regarding Item Development**

An iterative process was used to develop the initial item set of the Suicidal Desire Variability Scale (SDVS). First, the construct of interest was narrowly defined as the degree to which the SI facet of suicidal desire fluctuates. Fluctuation in the SI construct of suicidal desire (compared to intent or controllability of suicidal urges) was chosen given its unique prospective relationship with suicide attempts in adult psychiatric inpatients (Wang et al., 2021).

Six initial items were developed by the lead author, assuming a unidimensional construct (items 3, 4, 8, 11, 12, 13). All items were newly created for this measure; however, existing scales that measure similar constructs were also reviewed. For example, in creating the semantic structure of items to represent *variability* of a suicidal desire (not frequency or severity as most commonly assessed in SI-related self-report measures), validated scales that assess change in affective and emotional states were reviewed. Particularly, the Affect Liability Scale (Look et al., 2010) provided a validated measure that assesses rapid change in a psychological construct, and we mirrored that item structure (e.g., the use of a structure such as “one minute… and the next minute…”). To represent the construct of suicidal desire, we used language from previously utilized ambulatory items regarding suicidal desire (Kleiman et al., 2017), which we altered in two ways. First, Kleiman et al. (2017) used the term “intense suicidal desire.” However, focusing solely on intense suicidal desire could omit important information. Items were developed to ideally be void of language regarding the intensity of suicidal desire for item content to be clearly descriptive of *variability,* not intensity of suicidal desire. Given existing literature that minor wording changes to single suicide-related assessment items can produce discrepant endorsements even when items are presumed to assess the same construct (Ammerman et al., 2021), the initial item also used the term “want to kill myself” to provide another phrase that represented the construct of suicidal desire to determine if responding may differ based on this minor word alteration. Notably, items 11 and 12 assess suicidal desire variability by asking participants if suicidal desire was easily predictable (or not) because desire generally remained consistent (or did not) over the past week. These items were composed prior to the Coppersmith et al. (2024) study that demonstrated a tendency to overestimate future SI severity when adult participants are asked to prospectively predict their SI. Thus, the perceived accuracy of suicidal desire prediction as assessed in these two items may not solely represent how variable or consistent suicidal desire is, but a cognitive process of overestimation of future suicidal desire

The initial six items (items 3, 4, 8, 11, 12, 13) were read by one study co-author and two undergraduate research assistants for clarity, and minor wording changes were made. The six items were then run through an online Coleman–Liau index calculator to determine their readability, which returned a readability index of 6.87, indicating a reading level of approximately seventh grade. In consultation with the co-author, a decision was made to add additional items for initial exploratory analyses to allow enough items to potentially separate into different subscales according to item language. Specifically, if a subscale of “want” items were to be found separate from “desire” items, it was presumed that more items would be needed. Thus, additional items that use the phrasing of “how much I wanted to kill myself” versus “my desire to kill myself” were added. Similarly, additional items were added that start with “from one hour to the next” instead of a timeframe that focused on “one second…” to vary the timeframe of variability to determine if the timeframe resulted in changes to factor structure. Finally, three items that do not directly assess desire or want for suicide but simply “suicidal thoughts” were added to allow for tests of factor structure that could be further impacted by minor suicide-related content changes. This resulted in a set of 15 total items. These 15 items were presented to the entire team of co-authors, minor wording changes were made, and two additional items were added, resulting in the final set of 17 items tested in this investigation (Coleman–Liau index = 6.69). The study team consisted of three licensed clinical psychologists and four psychology doctoral students in clinical psychology.

**Supplementary Table S1**

*Study 1 SDVS Item Response Frequencies*

| **SDVS Item** | **Response Option *n* and %** | | | | | |
| --- | --- | --- | --- | --- | --- | --- |
|  | **1 (Strongly Disagree)** | **2**  **(Disagree)** | **3**  **(Somewhat Disagree)** | **4**  **(Somewhat Agree)** | **5**  **(Agree)** | **6**  **(Strongly Agree)** |
| 1 | **8** | **16** | 31 | 52 | 47 | 41 |
|  | **4.10%** | **8.20%** | 15.90% | 26.70% | 24.10% | 21.00% |
| 2 | **4** | **7** | **19** | 63 | 73 | 29 |
|  | **2.10%** | **3.60%** | **9.70%** | 32.30% | 37.40% | 14.90% |
| 3 | 35 | 67 | 41 | 29 | 17 | **6** |
|  | 17.90% | 34.40% | 21.00% | 14.90% | **8.70%** | **3.10%** |
| 4 | 23 | 53 | 29 | 53 | **29** | **8** |
|  | 11.80% | 27.20% | 14.90% | 27.20% | 14.90% | **4.10%** |
| 5 | 12 | 32 | 26 | 54 | 53 | **18** |
|  | **6.20%** | 16.40% | 13.30% | 27.70% | 27.20% | **9.20%** |
| 6 | **33** | 63 | 32 | 30 | 30 | **7** |
|  | 16.90% | 32.30% | 16.40% | 15.40% | 15.40% | **3.60%** |
| 7 | 18 | 38 | 33 | 41 | 49 | **16** |
|  | **9.20%** | 19.50% | 16.90% | 21.00% | 25.10% | **8.20%** |
| 8 | **30** | 54 | 41 | 31 | 33 | **6** |
|  | 15.40% | 27.70% | 21.00% | 15.90% | 16.90% | **3.10%** |
| 9 | 26 | 54 | 27 | 49 | 31 | **8** |
|  | 13.30% | 27.70% | 13.80% | 25.10% | 15.90% | **4.10%** |
| 10 | 25 | 51 | 36 | 36 | 33 | **14** |
|  | 12.80% | 26.20% | 18.50% | 18.50% | 16.90% | **7.20%** |
| 11 | 26 | 46 | 22 | 48 | 36 | **17** |
|  | 13.30% | 23.60% | 11.30% | 24.60% | 18.50% | **8.70%** |
| 12 | 23 | 63 | 37 | 38 | 30 | **4** |
|  | 11.80% | 32.30% | 19.00% | 19.50% | 15.40% | **2.10%** |
| 13 | 28 | 32 | 36 | 39 | 35 | 25 |
|  | 14.40% | 16.40% | 18.50% | 20.00% | 17.90% | 12.80% |
| 14 | 23 | 38 | 27 | 45 | 37 | 25 |
|  | 11.80% | 19.50% | 13.80% | 23.10% | 19.00% | 12.80% |
| 15 | 39 | 63 | 28 | 26 | 29 | 10 |
|  | 20.00% | 32.30% | 14.40% | 13.30% | 14.90% | **5.10%** |
| 16 | 25 | 43 | 26 | 50 | 34 | **17** |
|  | 12.80% | 22.10% | 13.30% | 25.60% | 17.40% | **8.70%** |
| 17 | **15** | **17** | 16 | 52 | 54 | **41** |
|  | **7.70%** | **8.70%** | 8.20% | 26.70% | 27.70% | 21.00% |

*Note*. SDVS = Suicidal Desire Variability Scale; Bolded values indicate a response endorsement < 10%.

**Supplementary Table S2**

*Study 1 SDVS Inter-item Correlations and Item Means and Standard Deviations*

| **Variable** | **1** | **2** | **3** | **4** | **5** | **6** | **7** | **8** | **9** | **10** | **11** | **12** | **13** | **14** | **15** | **16** | **17** |
| --- | --- | --- | --- | --- | --- | --- | --- | --- | --- | --- | --- | --- | --- | --- | --- | --- | --- |
| **1. SDVS 1** | ---- |  |  |  |  |  |  |  |  |  |  |  |  |  |  |  |  |
| **2. SDVS 2** | .54^**^ | ---- |  |  |  |  |  |  |  |  |  |  |  |  |  |  |  |
| **3. SDVS 3** | -.33^**^ | -.38^**^ | ---- |  |  |  |  |  |  |  |  |  |  |  |  |  |  |
| **4. SDVS 4** | -.06 | -.12 | .39^**^ | ---- |  |  |  |  |  |  |  |  |  |  |  |  |  |
| **5. SDVS 5** | .21^**^ | .30^**^ | -.40^**^ | -.52^**^ | ---- |  |  |  |  |  |  |  |  |  |  |  |  |
| **6. SDVS 6** | -.16^*^ | -.17^*^ | .46^**^ | .57^**^ | **-.73^**^** | ---- |  |  |  |  |  |  |  |  |  |  |  |
| **7. SDVS 7** | .24^**^ | .35^**^ | -.35^**^ | -.50^**^ | **.68^**^** | -.56^**^ | ---- |  |  |  |  |  |  |  |  |  |  |
| **8. SDVS 8** | -.01 | -.16^*^ | .43^**^ | **.69^**^** | -.57^**^ | **.67^**^** | **-.65^**^** | ---- |  |  |  |  |  |  |  |  |  |
| **9. SDVS 9** | -.03 | -.11 | .42^**^ | **.60^**^** | -.49^**^ | .55^**^ | -.52^**^ | **.72^**^** | ---- |  |  |  |  |  |  |  |  |
| **10. SDVS 10** | .30^**^ | .31^**^ | -.25^**^ | -.30^**^ | .43^**^ | -.32^**^ | .53^**^ | -.38^**^ | -.34^**^ | ---- |  |  |  |  |  |  |  |
| **11. SDVS 11** | .25^**^ | .31^**^ | -.28^**^ | -.26^**^ | .42^**^ | -.35^**^ | .59^**^ | -.40^**^ | -.35^**^ | .51^**^ | ---- |  |  |  |  |  |  |
| **12. SDVS 12** | -.15^*^ | -.20^**^ | .45^**^ | .45^**^ | -.44^**^ | .57^**^ | -.41^**^ | .59^**^ | .48^**^ | -.28^**^ | -.48^**^ | ---- |  |  |  |  |  |
| **13. SDVS 13** | .21^**^ | .32^**^ | -.38^**^ | -.32^**^ | .53^**^ | -.47^**^ | .56^**^ | -.48^**^ | -.38^**^ | .41^**^ | **.63^**^** | -.42^**^ | ---- |  |  |  |  |
| **14. SDVS 14** | .35^**^ | .37^**^ | -.38^**^ | -.28^**^ | .45^**^ | -.38^**^ | .45^**^ | -.38^**^ | -.37^**^ | .43^**^ | .56^**^ | -.33^**^ | **.66^**^** | ---- |  |  |  |
| **15. SDVS 15** | -.21^**^ | -.28^**^ | .31^**^ | .33^**^ | -.34^**^ | .37^**^ | -.37^**^ | .38^**^ | .38^**^ | -.23^**^ | -.29^**^ | .32^**^ | -.35^**^ | -.48^**^ | ---- |  |  |
| **16. SDVS 16** | .30^**^ | .30^**^ | -.23^**^ | -.15^*^ | .34^**^ | -.25^**^ | .32^**^ | -.17^*^ | -.16^*^ | .37^**^ | .45^**^ | -.18^*^ | .50^**^ | .50^**^ | -.16^*^ | ---- |  |
| **17. SDVS 17** | .37^**^ | .36^**^ | -.34^**^ | -.09 | .19^**^ | -.14 | .16^*^ | -.12 | -.11 | .24^**^ | .35^**^ | -.25^**^ | .35^**^ | .36^**^ | -.04 | .50^**^ | ---- |
| *M* | 3.05 | 3.31 | 1.86 | 2.26 | 2.78 | 2.04 | 2.59 | 2.13 | 2.24 | 2.28 | 2.42 | 2.10 | 2.51 | 2.55 | 2.01 | 2.43 | 3.08 |
| *SD* | 1.05 | 0.87 | 1.06 | 1.17 | 1.17 | 1.19 | 1.22 | 1.17 | 1.19 | 1.21 | 1.24 | 1.15 | 1.22 | 1.23 | 1.21 | 1.21 | 1.11 |

*Note*. SDVS = Suicidal Desire Variability Scale; The response options were collapsed into a response scale ranging from 1 to 4 (1 = Strongly disagree/Disagree, 2 = Somewhat disagree, 3 = Somewhat agree, 4 = Strongly agree/Agree); Bolded values indicate a correlation of .60 or greater.

***p* < .01. * *p* < .05.

**Supplementary Table S3**

*Study 2 SDVS Inter-item Correlations and Item Means and Standard Deviations*

| **Variable** | **1** | **2** | **3** | **4** | **5** | **6** | **7** | **8** | **9** | **10** | **11** | **12** | **13** | **14** | **15** | **16** | **17** |
| --- | --- | --- | --- | --- | --- | --- | --- | --- | --- | --- | --- | --- | --- | --- | --- | --- | --- |
| **1. SDVS 1** | ---- |  |  |  |  |  |  |  |  |  |  |  |  |  |  |  |  |
| **2. SDVS 2** | .48^**^ | ---- |  |  |  |  |  |  |  |  |  |  |  |  |  |  |  |
| **3. SDVS 3** | -.58^**^ | -.49^**^ | ---- |  |  |  |  |  |  |  |  |  |  |  |  |  |  |
| **4. SDVS 4** | -.38^**^ | -.46^**^ | **.64^**^** | ---- |  |  |  |  |  |  |  |  |  |  |  |  |  |
| **5. SDVS 5** | .51^**^ | .54^**^ | -.55^**^ | **-.71^**^** | ---- |  |  |  |  |  |  |  |  |  |  |  |  |
| **6. SDVS 6** | -.44^**^ | -.52^**^ | .59^**^ | **.78^**^** | **-.81^**^** | ---- |  |  |  |  |  |  |  |  |  |  |  |
| **7. SDVS 7** | .54^**^ | .48^**^ | -.54^**^ | **-.63^**^** | **.79^**^** | **-.69^**^** | ---- |  |  |  |  |  |  |  |  |  |  |
| **8. SDVS 8** | -.48^**^ | -.50^**^ | **.62^**^** | **.80^**^** | **-.78^**^** | **.85^**^** | **-.78^**^** | ---- |  |  |  |  |  |  |  |  |  |
| **9. SDVS 9** | -.51^**^ | -.45^**^ | **.63^**^** | **.78^**^** | **-.74^**^** | **.76^**^** | **-.73^**^** | **.87^**^** | ---- |  |  |  |  |  |  |  |  |
| **10. SDVS 10** | .52^**^ | .36^**^ | -.52^**^ | -.52^**^ | **.73^**^** | -.59^**^ | **.73^**^** | **-.66^**^** | **-.65^**^** | ---- |  |  |  |  |  |  |  |
| **11. SDVS 11** | .45^**^ | .36^**^ | -.44^**^ | -.46^**^ | **.63^**^** | -.49^**^ | **.66^**^** | **-.59^**^** | -.59^**^ | **.69^**^** | ---- |  |  |  |  |  |  |
| **12. SDVS 12** | -.51^**^ | -.43^**^ | .59^**^ | **.65^**^** | **-.66^**^** | **.66^**^** | **-.68^**^** | **.73^**^** | **.76^**^** | **-.66^**^** | **-.73^**^** | ---- |  |  |  |  |  |
| **13. SDVS 13** | .41^**^ | .42^**^ | -.39^**^ | -.47^**^ | .59^**^ | -.51^**^ | **.61^**^** | **-.61^**^** | -.59^**^ | **.60^**^** | **.68^**^** | **-.65^**^** | ---- |  |  |  |  |
| **14. SDVS 14** | .48^**^ | .43^**^ | -.48^**^ | -.48^**^ | **.61^**^** | -.53^**^ | **.60^**^** | **-.60^**^** | -.57^**^ | **.69^**^** | **.68^**^** | **-.65^**^** | **.74^**^** | ---- |  |  |  |
| **15. SDVS 15** | -.40^**^ | -.36^**^ | .43^**^ | .48^**^ | -.48^**^ | .52^**^ | -.47^**^ | .54^**^ | .52^**^ | -.48^**^ | -.46^**^ | **.61^**^** | -.53^**^ | **-.612^**^** | ---- |  |  |
| **16. SDVS 16** | .46^**^ | .39^**^ | -.41^**^ | -.42^**^ | .52^**^ | -.43^**^ | .55^**^ | -.52^**^ | -.52^**^ | **.62^**^** | **.64^**^** | **-.62^**^** | **.64^**^** | **.69^**^** | -.43^**^ | ---- |  |
| **17. SDVS 17** | .49^**^ | .45^**^ | -.44^**^ | -.47^**^ | .56^**^ | -.50^**^ | .55^**^ | -.58^**^ | -.51^**^ | .55^**^ | .53^**^ | -.58^**^ | .53^**^ | **.60^**^** | -.46^**^ | **.60^*^** | ---- |
| *M* | 2.83 | 3.22 | 2.20 | 2.33 | 2.75 | 2.17 | 2.53 | 2.37 | 2.37 | 2.37 | 2.25 | 2.54 | 2.25 | 2.30 | 2.33 | 2.24 | 2.85 |
| *SD* | 1.15 | 0.96 | 1.18 | 1.13 | 1.11 | 1.16 | 1.14 | 1.18 | 1.16 | 1.14 | 1.17 | 1.19 | 1.12 | 1.13 | 1.22 | 1.14 | 1.17 |

*Note*. SDVS = Suicidal Desire Variability Scale; The response options were collapsed into a response scale ranging from 1 to 4 (1 = Strongly disagree/Disagree, 2 = Somewhat disagree, 3 = Somewhat agree, 4 = Strongly agree/Agree); Gray items and values indicate items that were excluded from Sample 2 Confirmatory Factor Analyses. Bolded values indicate a correlation of .60 or greater.

***p* < .01. * *p* < .05.

**Supplementary Table S4**

*Study 2 SDVS Item Response Frequencies*

| **SDVS Item** | **Response Option *n* and %** | | | | | |
| --- | --- | --- | --- | --- | --- | --- |
|  | **1 (Strongly Disagree)** | **2**  **(Disagree)** | **3**  **(Somewhat Disagree)** | **4**  **(Somewhat Agree)** | **5**  **(Agree)** | **6**  **(Strongly Agree)** |
| 1 | 16 | 56 | 60 | 88 | 82 | 59 |
|  | 4.40% | 15.50% | 16.60% | 24.40% | 22.70% | 16.30% |
| 2 | **7** | **22** | 50 | 96 | 134 | 52 |
|  | **1.90%** | **6.10%** | 13.90% | 26.60% | 37.10% | 14.40% |
| 3 | 47 | 94 | 85 | 56 | 62 | **17** |
|  | 13.00% | 26.00% | 23.50% | 15.50% | 17.20% | **4.70%** |
| 4 | 39 | 76 | 85 | 88 | 52 | **21** |
|  | 10.80% | 21.10% | 23.50% | 24.40% | 14.40% | **5.80%** |
| 5 | **14** | 53 | 75 | 99 | 92 | **28** |
|  | **3.90%** | 14.70% | 20.80% | 27.40% | 25.50% | **7.80%** |
| 6 | 42 | 104 | 77 | 69 | 57 | **12** |
|  | 11.60% | 28.80% | 21.30% | 19.10% | 15.80% | **3.30%** |
| 7 | **28** | 67 | 74 | 98 | 63 | **31** |
|  | **7.80%** | 18.60% | 20.50% | 27.10% | 17.50% | **8.60%** |
| 8 | **35** | 86 | 71 | 83 | 68 | **18** |
|  | **9.70%** | 23.80% | 19.70% | 23.00% | 18.80% | **5.00%** |
| 9 | 37 | 82 | 71 | 90 | 66 | **15** |
|  | 10.20% | 22.70% | 19.70% | 24.90% | 18.30% | **4.20%** |
| 10 | 44 | 71 | 74 | 95 | 54 | **23** |
|  | 12.20% | 19.70% | 20.50% | 26.30% | 15.00% | **6.40%** |
| 11 | 53 | 85 | 69 | 81 | 58 | **15** |
|  | 14.70% | 23.50% | 19.10% | 22.40% | 16.10% | **4.20%** |
| 12 | **33** | 70 | 69 | 81 | 75 | **33** |
|  | **9.10%** | 19.40% | 19.10% | 22.40% | 20.80% | **9.10%** |
| 13 | 52 | 75 | 78 | 93 | 50 | **13** |
|  | 14.40% | 20.80% | 21.60% | 25.80% | 13.90% | **3.60%** |
| 14 | 50 | 77 | 67 | 100 | 47 | **20** |
|  | 13.90% | 21.30% | 18.60% | 27.70% | 13.00% | **5.50%** |
| 15 | 39 | 95 | 67 | 67 | 62 | **31** |
|  | 10.80% | 26.30% | 18.60% | 18.60% | 17.20% | **8.60%** |
| 16 | 58 | 75 | 76 | 85 | 50 | **17** |
|  | 16.10% | 20.80% | 21.10% | 23.50% | 13.90% | **4.70%** |
| 17 | 36 | 40 | 48 | 92 | 104 | 41 |
|  | 10.00% | 11.10% | 13.30% | 25.50% | 28.80% | 11.40% |

*Note*. SDVS = Suicidal Desire Variability Scale; Gray items and values indicate items that were excluded from Sample 2 Confirmatory Factor Analyses. Bolded values indicate a response endorsement < 10%.

**Supplementary Table S5**

*Study 2 SDVS Confirmatory Factor Analyses Item Loadings*

| **SDVS Item** | **9-item Models** | | |  | **5-item Dynamic Model** |  | **4-item Static Model** |
| --- | --- | --- | --- | --- | --- | --- | --- |
|  | **One Factor** | **Two Factors** | |  | **One Factor** |  | **One Factor** |
|  |  | **1** | **2** |  |  |  |  |
| 1 | .69* | .71* | ---- |  | .66* |  | ---- |
| 3 | -.78* | ---- | .80* |  | ---- |  | .80* |
| 4 | -.86* | ---- | .87* |  | ---- |  | .92* |
| 6 | -.88* | ---- | .90* |  | ---- |  | .92* |
| 10 | .85* | .88* | ---- |  | .87* |  | ---- |
| 11 | .86* | .89* | ---- |  | .87* |  | ---- |
| 12 | -.90* | ---- | .95* |  | ---- |  | .83* |
| 14 | .86* | .88* | ---- |  | .89* |  | ---- |
| 16 | .80* | .82* | ---- |  | .84* |  | ---- |

*Note*. SDVS = Suicidal Desire Variability Scale; 9-item two-factor model factor correlation *r* = -.87, *p* < .001; 9-item Omega Internal Consistency = .91; Dynamic Items Omega Internal Consistency = .88; Static Omega Internal Consistency = .88.

* *p* < .05.

**Supplementary Table S6**

*Study 3 Correlations between Metrics of Suicidal Desire Change Measured During the Ambulatory Phase and SDVS Items*

| **Variable** | **Desire RMSSD** | **Want RMSSD** | **1 *SD* Change in Desire** | **1 *SD* Change in Want** |
| --- | --- | --- | --- | --- |
| How much I wanted to kill myself did not change very often | -.43** | -.47** | .01 | -.14 |
| How much I wanted to kill myself stayed about same from one hour to the next | -.22 | -.24 | -.16 | -.15 |
| From one hour to the next, my thoughts of suicide did not change | -.23 | -.27 | -.04 | -.13 |
| My desire to kill myself was fairly predictable because it did not change much | -.25 | -.30 | -.07 | -.04 |
| How much I wanted to kill myself went up and down a lot | .29 | .41* | -.10 | .09 |
| How much I wanted to kill myself changed multiple times from one hour to the next | .30 | .26 | -.14 | .13 |
| My desire to kill myself was mostly unpredictable because it changed so quickly | .26 | .26 | -.15 | .06 |
| I frequently switched from wanting to kill myself to not wanting to kill myself | .33* | .36* | -.28 | -.04 |
| Many times, I quickly went from not wanting to kill myself to really wanting to kill myself | .46** | .55*** | .02 | .18 |

**p* < .05, ***p* < .01, ****p* < .001. SDVS = Suicidal Desire Variability Scale; RMSSD = root mean square of successive differences
